# Probiotics in Counteracting the Role of Neutrophils in Cancer Metastasis

**DOI:** 10.3390/vaccines9111306

**Published:** 2021-11-10

**Authors:** Upasana Mangrolia, Jabez W. Osborne

**Affiliations:** School of Bio Sciences and Technology, Vellore Institute of Technology (VIT), Vellore 632014, India; upasanamangrolia@gmail.com

**Keywords:** neutrophils, NETs, cancer, metastasis, probiotics

## Abstract

Neutrophils are known for their role geared towards pathogen clearance by different mechanisms that they initiate, primarily by the release of neutrophil extracellular traps (NETs). However, their immune-surveillance capacity accompanied with plasticity in existing as interchangeable subsets, discovered recently, has revealed their property to contribute to complex cancer pathologies including tumor initiation, growth, angiogenesis and metastasis. Although there is a growing body of evidence suggesting a critical balance between the protumoral and antitumoral neutrophil phenotypes, an in-depth signaling pathway analysis would aid in determination of anticipatory, diagnostic and therapeutic targets. This review presents a comprehensive overview of the potential pathways involved in neutrophil-triggered cancer metastasis and introduces the influence of the microbial load and avenues for probiotic intervention.

## 1. Introduction

Although malignant tumors are predominated by the presence of cancer cells, they also include a range of normal cells capable of influencing the growth and displacement of cancer cells to distant parts of the body. Interestingly, among these noncancerous cells, immune cells cater to promoting the survival and spread of tumors despite being otherwise educated to eradicate them. The possible underlying reasons include their uncontrolled infiltration in the tumor microenvironment and/or their derailed functioning [1].

The role of the immune system in cancer progression has been receiving extensive attention, especially in the context of neutrophils. Neutrophils are the most abundant white blood cells, and their accumulation in tumor sites has been associated with poor patient prognosis. With the discovery of neutrophil subtypes, their phenotype and role in tumors appear to be partially directed by cancer cells [1].

Thus, comprehensively exploring the mechanism is crucial for understanding the pathophysiological aspects and identifying the subsequent targets. These emerging complex targets call for counter convoluted remedies, one of which can be probiotics. At present, the International Scientific Association for Probiotics and Prebiotics (ISAPP) defines probiotics as “live microorganisms that, when administered in adequate amounts, confer a health benefit on the host”. Neutrophils play a role in cancer metastasis via different pathways (e.g., inflammation, immune cell survival status and modulation, angiogenesis, vascular permeability), which have been interestingly explored independently as probiotic targets. In a comprehensive look in the context of connecting probiotics and neutrophil activity, although limited literature is available at present, broad data on the use of probiotics in intervening in neutrophil-mediated metastasis are presented in this review.

## 2. Immune System Keeping Homeostasis “In Check”

The neutrophils are a part of the innate immune system which also includes eosinophils, basophils, mast cells, dendritic cells (DCs), natural killer cells (NKs) and macrophages. These function in harmony to mount an initial defense by recognizing and attempting to destroy pathogens and abnormal cells such as cancer cells, while also triggering a danger signal to other cells and tissues [2,3]. Thus, during an upsurge inflammatory response. It is important to acknowledge the bystander effect of cytotoxic chemicals on nonpathogenic tissue, extracellular matrix and immune cells. However, the immune cells, while being attached to infection or tissue injury sites by pathogen-associated molecular patterns (PAMPs) or damage-associated molecular patterns (DAMPs), can respond to signals from abnormal host cells upon prolonged exposure or delayed restoration. A long-term failure in resolving inflammation after infection can thus result in aberrant immune signaling, gene and protein modifications, extensive tissue damage and ultimately carcinogenesis [4]. Further, the immune system’s capacity to impart “immunoediting” in the course of cancer was presented by Dun et al. in 2004 [5]. According to this theory, the immune response to the neoplasm is formed (asymptomatic) where the immune system eliminates the possible tumorigenic cells [6,7]. This identification and extermination of the tumor cells are termed immune surveillance [8]. However, a prolonged fight may lead to sustained cancer in equilibrium with immune cells, with an emerging favorable environment and absence of any distinct symptoms [7,9,10]. This is followed by the last phase where the immune cells receive instructions from the cancer cells to effectively deregulate, block and escape the immune system and their presence is reflected in progressing damage and clinical detectability. There is an increasing body of evidence supporting the role of immune cells beyond the host’s mechanism of fighting off cancer [9,10,11,12], suggesting the intervention of reprogrammed immune cells in enabling tumor growth [13]. Unlike the other immune cells, such as T lymphocytes, dendritic cells, natural killer cells and macrophages, neutrophils have been only recently considered for their role in cancer and are emerging as an important factor in every stage of cancer.

## 3. Neutrophils: Short-Lived Intense Players of Immunity

**Figure 1:**
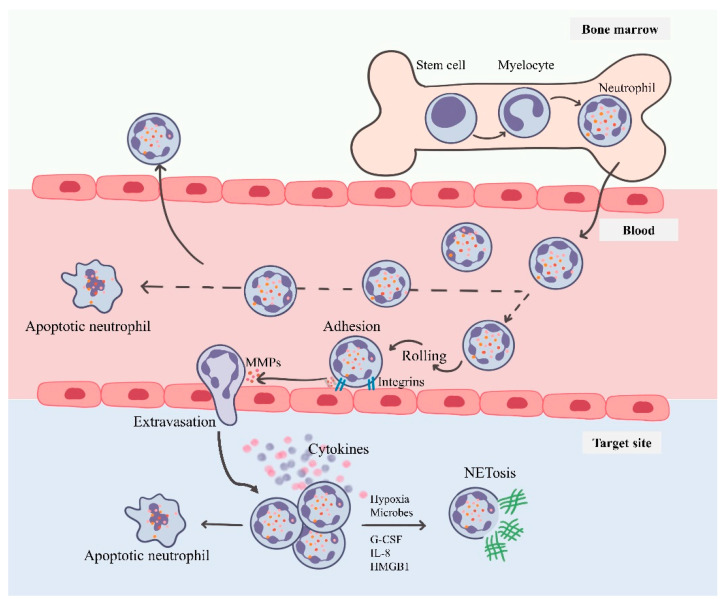
Neutrophils—from bone marrow to target site: The neutrophils originate from the bone marrow with a short lifespan in circulation. They migrate towards the chemokines following a rolling–tethering–extravasation step. These activated neutrophils both target the infectious agent and further undergo programmed cell death or under tumor-like microenvironment (cancer cells, hypoxia, stroma cell signals) are triggered to undergo NETosis.

Neutrophils constitute about 80% of the white blood cells but are short-lived, which has masked their role in cancer until recently. Neutrophils emerge from the bone marrow in the terminally differentiated state and circulate in the bloodstream with the primarily known purpose of antimicrobial activity. Neutrophils are phagocytes containing chemicals and proteins in their granules (azurophilic, primary; specific, secondary; gelatinase, tertiary), facilitating the internalization of microbes in phagosomes [14]. Their migration is dependent on a variety of chemoattractants originating from injured tissue, pathogenic stimuli, other immune cells or tumors. These chemoattractants can be broadly classified into four groups, namely chemokines, N-formylated peptides, complement anaphylatoxins and lipids, which interact through G-coupled protein receptors (GPCRs) [15]. Upon arriving at the target site, neutrophils secrete β2-integrin-extruding vesicles near the cell surface [16], enabling their tethering. They further secrete matrix metalloproteinases (MMPs) and other oxidants which degrade the endothelial wall, hence allowing the passage into the inflammatory site [17]. The neutrophil-derived oxidants play a key role in the elimination of infectious agents, but under chronic inflammatory conditions, they can cause damage to the host tissue by damaging the DNA and/or impart epigenetic modifications that result in deregulated gene expression which can extend up to abnormal cell division. At the inflamed tissue site, the neutrophils are activated to secrete the cytokines, defensins and stimulatory factors via degranulation, thereby modulating the local tissue environment and recruiting subsequent immune cell cascade (Figure 1) [17].

Under neutropenic condition, as observed in cancer patients upon chemotherapy and radiotherapy [18], when patients are administered the growth factor granulocyte colony-stimulating factor (G-CSF), it can stimulate the de novo neutrophil generation via bone marrow hematopoiesis [19]. Detailed follow-up in patients treated with G-CSF has revealed the onset of pathological conditions accelerating the metastasis [20,21,22,23].

## 4. Neutrophils in Orchestrating Cancer

**Figure 2:**
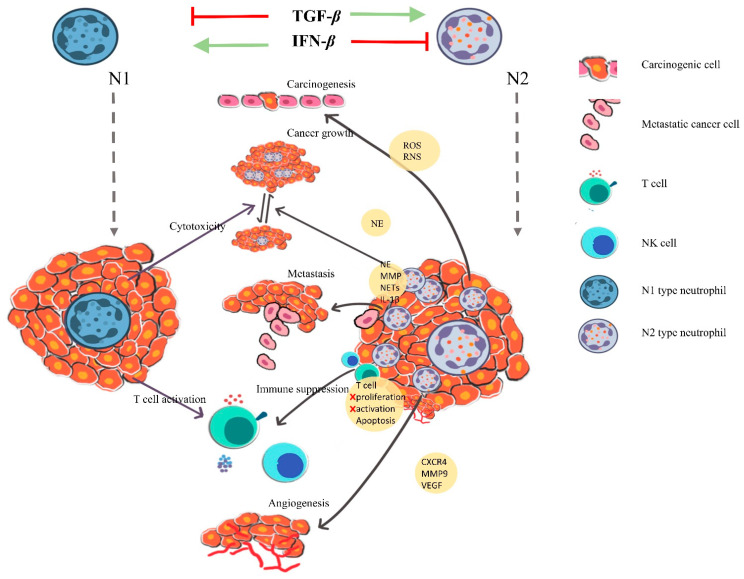
Role-dependent differentiation of neutrophils (N1 and N2) in cancer: The polarization of neutrophils is induction-dependent, IFN-β for N1 and TGF-β for N2. N1 neutrophils are categorized as antitumor neutrophils based on their cytotoxicity and T cell activation characteristics. N2 neutrophils are protumor neutrophils that induce carcinogenesis via ROS and RNS pathway; impart cancer growth directed by neutrophil elastases, the MMPs, NETs, NE and IL-1β; and promote cancer cell metastasis. N2 phenotype also suppresses T cell activation and proliferation, induces T cell apoptosis and inhibits NK cell activity; N2-type cells also participate in angiogenesis via CXCR4, MMP9 and VEGF expression modulation.

The tumor-associated neutrophils (TANs) are suggested to express either of two morphologies: N1 (antitumor) and N2 (protumorigenic). The cytotoxicity of neutrophils on tumor cells is imparted via ROS or antibody-dependent cellular cytotoxicity which stimulates T cells and dendritic cells, further activating other immune components. The N2 phenotype promotes tumor invasion and metastasis; enforcement of angiogenesis; and expression of epidermal growth factor (EGF), transforming growth factor β (TGF-β), platelet-derived growth factor (PDGF) and vascular endothelial growth factor (VEGF) [24,25]. A similar pattern of expression is also observed in inflammatory conditions, and the tumor microenvironment is inclined towards a chronically active inflammatory condition which provides a more conducive state for tumor growth when compared to the acute state [26]. This is derived from the fact that leukocytes at chronic inflammatory sites produce growth factors that promote proliferation (TNF-α, TGF-β, EGF) [27,28,29,30], along with histamine and heparins [31], ECM-modulating proteases; additionally, tumor cells and activated leukocytes produce ROS in abundance, leading to oxidative stress, thereby stimulating the modulation of genetic and epigenetic factors (Figure 2) [32].

It has been reported that TGF-β induces N2 phenotype in TANs [33,34]; when present near tumors, these TANs are observed to release cytokines capable of initiating a positive feedback loop, thereby recruiting a greater number of neutrophils to the tumor site [35]. These TANs not only promote tumor growth or support invasion by angiogenesis, but also mobilize and accumulate in future tumor metastasis sites, i.e., the “premetastatis niche”, and prepare the microenvironment [36,37], as is explained in detail in further sections.

Additionally, myeloid-derived suppressor cells (MDSCs), a group of pathologically activated and systematically expanded immature myeloid cells, are highlighted as being present in tumor-bearing hosts. The neutrophils constitute a part of the polymorphonuclear MDSC (PMN-MDSC) subset and are known to promote immunosurveillance evasion, premetastatic niche formation, EMT and angiogenesis [38,39,40]. MDSC-type neutrophils can highly express inducible nitric oxide synthase (iNOS) and arginase which are involved in the main mechanism for T cell suppression [41,42]; they also induce T cell apoptosis and block the T cell activation as well as effector functions [41,42]. In breast cancer model and ex vivo studies, NK cells are reported to govern the tumor modulatory effects of the neutrophils, where neutrophils, being tumoricidal, are shown to suppress the tumoricidal activity on NK cells. Here, high neutrophil expression suppressed NK cells and subsequently increased metastasis, indicating that although neutrophils and NK cells both have tumoricidal activity, NK cells have higher tumoricidal activity which can eventually downregulate metastasis. Interestingly, in the absence of NK cells, low and not high neutrophil infiltration promoted metastasis [43]. Further, neutrophil extracellular traps (NETs) have been recently found to play a role in providing a physical barrier that shields the tumor cells from interacting with the nearby antitumor immune cells such as CD8+ T cells and NK cells [44,45].

### 4.1. Cancer Also Promotes Neutrophil Activation

Signals in the form of granulocyte colony-stimulating factor (G-CSF), produced by several tumors themselves, also play a role in increasing neutrophils and inducing their activation [46,47]. The cancer-associated fibroblasts (CAFs), known for tumorigenic activity, produce a protagonist peptide molecule, amyloid β, which can drive NET release via an ROS-dependent pathway [48]. The hyaluronan produced by tumor cells can interact with TLR-4 receptors on neutrophils and induce long-lived neutrophils, which further promotes contact-dependent cancer cell motility; however, this can be blocked by inhibiting the activation of PI3K/Akt signaling in neutrophils, since PI3K/Akt signaling has been implicated in regulating the proinflammatory cytokine expression, thus presenting a potential therapeutic target [49].

### 4.2. NETs in Mediating Cancer

**Figure 3:**
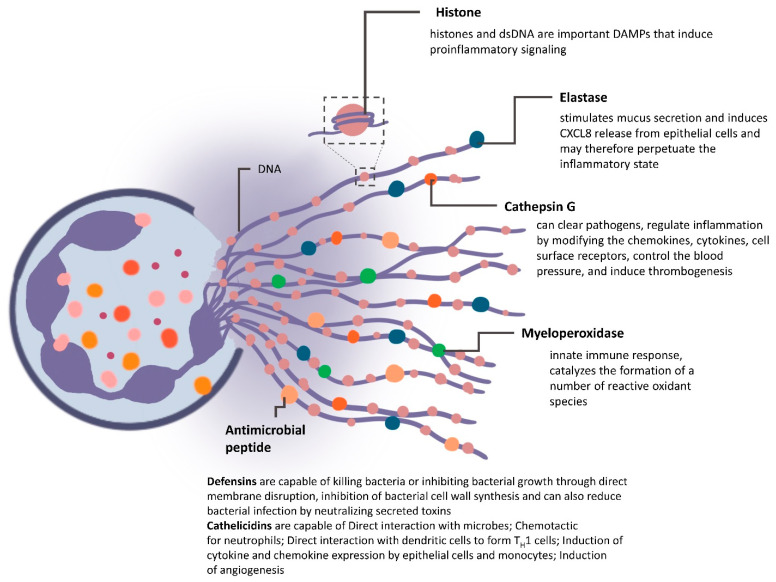
Structure of neutrophil extracellular traps: upon receiving the appropriate stimuli, neutrophils release extracellular DNA traps which are overall composed of histone-decorated DNA strands and proteases, including elastase and cathepsin G.

Although neutrophils are reported to play a negative role in the aggravated inflammatory process, this has been lately attributed to NETs [50,51,52,53,54]. Neutrophils can release histone-bound nuclear DNA along with cytotoxic granules as NETs. NETs have been identified as an element of the nonspecific immunity that affirms their response by causing the microorganisms to stagnate, thereby preventing their spread and ensuring a high localized concentration of antipathogenic factors. These NETs were first identified by Berger et al., who demonstrated the capacity of tumors to form NETs in Ewing’s sarcoma patients [34]. Neutrophil stimulation with phorbol myristate acetate (PMA) showed a web-like release of DNA structures coated with histones, elastase, cathepsin G and myeloperoxidase (MPO) (Figure 3) [34]. Overall, 20 different proteins are thought to be present in a given NETome. Subsequently, it was shown that NET release was associated with neutrophil rupturing, leading NETosis to be characterized as programmed neutrophil death [51]. Unlike apoptosis and necrosis, NETosis is ROS-dependent, which is a result of reduced nicotinamide adenine dinucleotide phosphate (NADPH) oxidase [51,55]. However, it has been demonstrated that naturally induced NET formation can occur independent of cell membrane lysis [56,57] and occurs via TLR4 activation [58]. Here, the mitochondrial DNA is released [59]; mitochondrial DNA also lacks, by definition, histones. Detailed studies highlight the positive and negative effects of NETs on tumor cells. The NET component MPO has been reported to inhibit and kill melanoma cancer cells. Histones, an important part of the NETs, are capable of destroying the epithelial cells of the blood vessels that feed the tumors. Further, defensins in the NETs mediate tumor cell lysis, recruit dendritic cells and have antiangiogenic properties [29,30,36,48]. MMP-9 has been previously presented as a modulator of innate immune response in antitumor activity [60] in which MMP-9-dependent neutrophil infiltration surfaced as an antitumor process, which can possibly be via N1 phenotype recruitment. Conversely, the TANs and NETs were exclusively present in patients with metastasis and in patients with relapse after intense chemotherapy. Moreover, NETs promote metastasis by degrading the extracellular matrix (ECM) and releasing protease components such as matrix metalloproteinase 9 (MMP-9) that block tumor apoptosis. MMP-9 potentially has been reported to promote angiogenesis and neovascularization within the tumor. Thus, NETs can overall promote migration, invasion and metastasis, as evidently reported in lung cancer cells [31,32]. Since NETs form a physical barrier between the tumor and immune cells, their potential in inhibiting immune cell defense too must not be overlooked.

### 4.3. NETs in Metastasis

**Figure 4:**
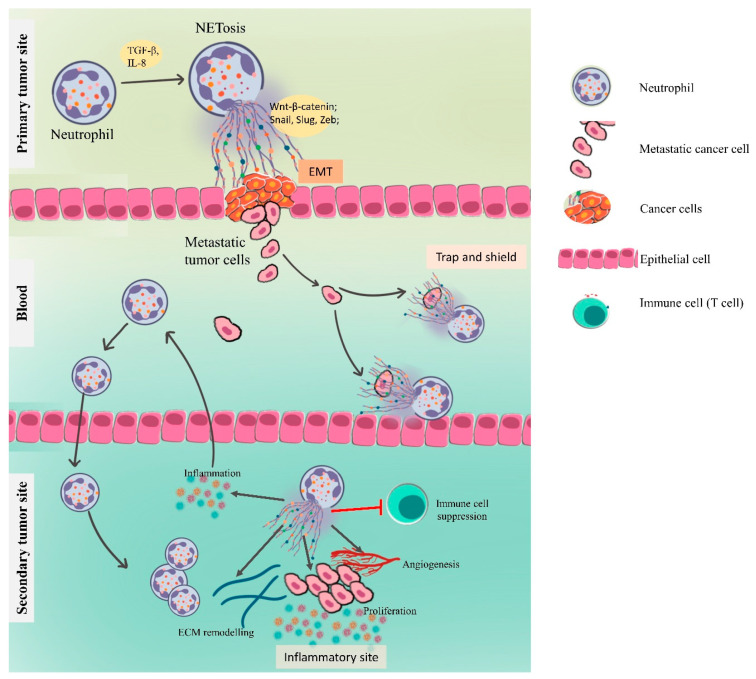
Neutrophil extracellular traps (NETs) induce cancer metastasis: At the primary cancer site, NETs can awaken the dormant cancer cells and can promote epithelial–mesenchymal transition (EMT). Once in the circulation, these circulating cancer cells (CTCs) can become entrapped in the circulating NETs and be protected from immune cells; at the same time, they are exposed to high concentrations of local neutrophil elastase with other factors that can aid in proliferation. The NETs can prepare the secondary/metastatic tumor site, i.e., inhibit immune-surveillance by T cells and NK cells, release MMP-9 and serine proteases which promote angiogenesis via VEGF, modulate extracellular matrix (ECM) in favor of cancer proliferation and recruit inflammatory cytokines which further attract more neutrophils.

Since neutrophils are a part of the connective tissue (blood), their migration from one organ to another is not unexpected. Moreover, manipulation of tumors during surgery can result in an increased number of free cancer cells in the blood. Thus, the adhesion of cancer cells to neutrophils is of concern since they can act as carriers, which is in accordance with the previously described ability of the neutrophils to migrate to a distant metastatic niche. Interestingly, neutrophils are observed to exert effects on the most rate-limiting stage of the metastatic process: the organ colonization step [21,61]. A study on mice reported the inhibition of lung cancer cell adhesion to PMA-stimulated neutrophils after treatment with DNases [62]; similar reports have been made for lung and pancreatic cancer [63]. This indicates that the neutrophils and/or NETs present in these distant metastatic sites (microvessels) [64] may capture the circulating cancer cells with DNA strands (an important component of NETs). Similarly, understanding the emerging role of NETs in promoting cancer metastasis can open avenues for a more specific or targeted therapeutic. The potential target-pathways include the role of neutrophils and NETs in epithelial–mesenchymal transition (EMT), cancer cell adhesion and premetastatic niche modulation (inflammation, tumor immune evasion, angiogenesis and vascular permeability and awakening the dormant cancer cells) (Figure 4) [65].

#### 4.3.1. NETs Driving the Epithelial–Mesenchymal Transition

Several reports have ascertained that neoplastic cells inherit the ability to invade the local microenvironment and seed in secondary sites. In their journey, however, they encounter several obstacles, including immune surveillance, nutrient/metabolic stress and crossing the endothelial barrier before seeding at the target site. This requires a facilitated translocation which is armed by the immune cells, among which NETs are an emerging player in metastasis. The NETs isolated from PMA-induced human neutrophils in vitro have been reported to cause loss of epithelial junction, promote the development of mesenchymal phenotype and induce aberrant activation of the Wnt/β-catenin signaling pathway in normal epithelial cells [66]. Similar results in neoplastic epithelial cells and in murine models with higher expression of EMT regulators (Snail, Slug and Zeb) along with EMT stimulants (TGF-β and IL-8) have been observed [67,68]. Thus, the ability of NETs to initiate EMT in normal and neoplastic cells suggests their early contribution to metastasis. This is further affirmed in a report where the invasive and migratory characteristics of breast cancer cells were partially impacted upon DNase treatment [67]. Here, in MCF-7 cells, digestion of NETs with DNase did not significantly impact tumor cell migration or MMP9 gene expression, rendering DNA integrity to be dispensable in the effect of NETs over MCF-7 cells [67]. Further, NET-associated elastase or NET-derived HMGB1 inhibition showed abrogation of the EMT stimulation [68]. Collectively the data suggest the role of NET proteins in the EMT initiation; however, since NET-DNA seems to be involved in the EMT process, DNA might contribute to a different metastatic cascade.

#### 4.3.2. Cancer Cell Adhesion

The cancer cells entering the circulating system upon EMT induction are termed circulating tumor cells (CTCs) and have been reported to be present in high amounts in patients [61]. These cells are under constant pressure from the fluid shear forces, oxidative stress and immunity, resulting in few of them entering the micrometastasis state [61]. The NETs accumulated in distant organs are shown to influence the recruitment of CTCs via chemotaxis, as observed in liver metastasis in breast cancer and colon cancer patients where coiled-coil domain-containing protein 25 (CCDC25), a transmembrane protein, acts as an attractant of the NET-associated chemotactic factors [69] and also initiates the β-parvin–RAC1–CDC42 cascade further supporting cytoskeleton rearrangement and directional migration of tumor cells [69]. In the CTC state, the cancer cells must be recognized and sequestered by NETs as a defense appliance, but conversely, NET entrapment advances CTC invasiveness and metastatic potential [70]. Possible mechanisms experimentally deduced are inflammatory signal activation via NET-TLR4-mediated NF-κB [70], STAT3 and MAPK/p38 pathway [71]; here, TLR4 stimulation induces NF-kB-mediated expression of inflammatory cytokines via STAT3 and MAPK/p38 pathway. Additionally, mechanical entrapment in NET-DNA [62] and integrin-mediated adhesion [72] are also some of the possible tethering mechanisms.

#### 4.3.3. NETs in Preparing the Premetastatic Niche

With the immunological and metabolic vulnerability, organotropism of cancer cells requires a hospitable environment at the target organ sites. This premetastatic niche results cumulatively from tumor cells, host stromal cells and bone marrow-derived cells, with a recent focus being neutrophils in metastatic cases of liver [65] and lung [73], partially mediated via NET-remodeled microenvironment; this is described in Figure 4, at the secondary tumor site.

##### Inflammation and NETs

Inflammation is among the key signs of the premetastatic niche, and the role of NETs in these sites is emerging evidently. The inflammatory milieu provides a seeding environment for the disseminated tumor cells and promotes subsequent survival and proliferation. In colorectal cancer condition, elevated levels of neutrophils at the primary tumor were correlated with the enhanced NETs in the liver, which also induced proinflammatory cytokine (IL-8, IL-6 and TNF-α) production in trapped cancer cells [74]. The hence released TNF-α has been shown to promote EMT [75]. Overall, the neutrophil–NET–inflammation cycle remains a positively regulated cycle promoting cancer metastasis.

##### Angiogenesis and Vascular Permeability

Since angiogenesis and vascularization are mediums of nutrients, their enhancement becomes a prerequisite for metastatic colonization. Although evidence on the role of NETs in angiogenesis and vascular permeabilization in the context of cancer is limited, their role in tissue repair [76], pulmonary hypertension [77] and corneal neovascularization [78] has been functionally linked to angiogenesis. The possible mechanisms include endothelial cell activation, i.e., TLR-4/NF-κB signaling [77] or inflammation-induced VEGF upregulation [78,79]. Additionally, NETs have been recognized for their role in clearing senescent endothelial cells [80] and disrupting the endothelial barrier, possibly achieved via neutrophil proteases including elastase and MMP [81,82]. Their role in angiogenesis and vascular permeabilization is overall supportive in cancer metastasis.

#### 4.3.4. NETs in Awakening the Dormant Cancer Cells

The plasticity of cancer cells as reflected in their dormant state assists their evasion from the immune system and radiological radar. The disseminated tumor cells, upon reaching the metastatic site, usually undergo dormancy, but over a period of time, they exit the senescence state and resume functionality resulting in metastatic tumors [83]. While resuming their role, the T cells and NK cells mediate their elimination in a programmed manner, thus preventing their relapse [84]. NETs have been unveiled for their role in dormant cell reactivation, mediated by NET-associated proteases [85].

Apart from identifying neutrophils for their role in cancer, it is equally important to address the question of whether neutrophil and NET inhibition is worth the risk when weighed against the chances of sepsis. Additionally, equally promising infection-controlling agents such as antibiotics are readily available; however, their side effects preclude their rational use. Overall, with advancing times, the treatment methods have been enhanced, and so have the possible side effects. Although the options of targeted therapy and immunotherapy are available, conventional chemotherapy is a requirement in case of advanced and recurrent cancer conditions [86]. The chemotherapeutics are commonly known for their side effects such as nausea, diarrhea and vomiting [87,88]. Such side effects are detrimental in terms of nutritional loss and hampered immune system function leading to a delayed treatment cycle, increasing the suffering period and cost [89,90,91].

There is an increase in evidence-based inclination toward the use of probiotics in relieving cancer pathologies, one being gut microbiome restoration (and pathogen elimination) and the other being immune modulation which can promote cancer inhibition. Despite several studies, the explicit role of NETs in cancer still remains elusive. However, with the key components identified in the NETs, selectively targeting the pathology-promoting factors can be a promising approach. With emerging data, probiotics have been identified to counteract the tumor-promoting factors, irrespective of the study being carried out with NETs as the central target.

## 5. Probiotics as a Potential Therapeutic

The use of bacteria in cancer immunotherapy can be traced back to 1976, when Bacillus Calmette-Guérin (BCG) was first intravesically instilled in bladder cancer patients and was overall successful [92,93]. However, BCG instillation was further found to have side effects including sepsis, organ failure and even disseminated infections [94,95], thereby requiring the discovery of bacteria with similar activity and lesser side effects. A depletion of the microbiota has been correlated with lowered systemic peptidoglycan concentrations, as deduced by less killing of infectious agents (*S. pneumoniae* and *S. aureus*) by bone marrow-derived neutrophils. These data thus showed a mechanism for microbiota-mediated systemic immunomodulation, demonstrating that translocated microbial products and their modulation by probiotics can be beneficial for the host in controlling bone marrow myelopoiesis and thereby enhancing systemic innate immune function [96]. Considering the role of neutrophils in almost all steps of cancer metastasis which is exerted in response to tumor-derived incitements [97], the inhibition of their function by probiotics might be an efficient strategy that impedes metastasis. It is important to understand the relationship between the microbiome and the neutrophils to probe probiotic interventions in the desired direction.

### 5.1. Overcoming Inflammatory Cytokines with Probiotics

Trained immunity is an outcome of a series of nonspecific insults, preparing for protection under secondary points of infection/alterations [98,99]. Despite the probiotic effects on the neutrophil function, the short life span of the neutrophils raises the question of long-term adaptation of neutrophils to similar conditions. On the contrary, this works in the favor of the system, since the effect and activation can thus be regulated. Early investigations have shown that neutrophils can be primed by cytokines such as IL-6, IL-8 and TNF-α, which are regulated by the microbiome [100,101]. Thus, a healthy microbiome from an early stage in life can promote an incessant granulopoiesis by keeping inflammation under control. Additionally, this microbiome can be assisted by probiotics; for instance, a constituent of polysaccharide peptidoglycan complex on *Lactobacillus casei* strain Shirota (*LcS*) has been shown to exert beneficial effects in a murine model of inflammatory bowel disease and colitis-associated cancer through inhibition of IL-6/STAT3 signaling (via TLR4 pathway) [97]. Further, with respect to various innate immune cells, the proinflammatory activity of the neutrophils in terms of tissue infiltration, phagocytosis and NET formation elevates with age (from birth to early adult) and is in particular regulated by the TLR4/MyD88 pathway [102]. This has been correlated with the microbiome population, in a study confirming the effect of microbiota composition on neutrophil properties [102].

Similar to cancer cells, infection by pathogens such as enterotoxigenic Escherichia coli (ETEC) K88 has been observed to induce chemokine-mediated neutrophil migration, possibly via IL-8, GRO-a and ENA-78 release, that further leads to CAC pathology [103]. Interestingly, *B. animalis* or *LGG* treatment on Caco-2 cells could result in only a low level of neutrophil migration [104], supporting the possible application of these treatments in limiting inflammation-induced neutrophil recruitment.

### 5.2. Probiotics and Immune Cell Death

Neutrophils that age gradually not only enter senesce but also impart reduced chemotaxis, exhibiting reduced phagocytic activity and enhanced superoxide generation prior to entering the apoptotic phase in the elderly [105]. Their longer presence can impede the average neutrophil function. This can be overcome with strategic probiotic administration. Daily consumption of *B. lactis* Bi-07 has been shown to improve the phagocytic activity of monocytes and granulocytes in healthy adults (elderly) [106]; this can be considered as the future basis of considering and designing experiments that explore similarly improved phagocytic activity in neutrophils. *Lactobacillus helveticus* supplementation in vivo increased activity of neutrophil enzymes NADPH oxidase (NOX2) and MPO and moreover maintained the increased levels for a longer duration of 2 months [107]. Park et al. evidently proved the activity of neutrophil NOX2 in causing invasion-promoting NET formation [108]. The study importantly concludes that although probiotic stimulation provokes neutrophil activity, it does not stimulate the innate immune system upon reaching a threshold. This enhancement in the cellular activities to a stable maximum is important since hyperactivation of the immune system can result in unwanted consequences [107]. In contrast to *B. lactis* Bi-07, it is equally important to scrutinize individual probiotic strains for their activity on phagocytic activity of neutrophils since *L. johnsonii* La1, also a probiotic, is reported to reduce the phagocytic activity of the neutrophils [109,110]. However, neutrophils incapable of function-reversibility must commit to the death cycle to improve the general output of the immune system.

Neutrophils are abundant in intestinal inflammatory conditions; they are recruited to the site of inflammation as the primary response and can easily cross the epithelium to enter the intestinal lumen. As mentioned earlier, excessive neutrophil infiltration into the mucosa can potentiate severe tissue damage. This overall indicates the need for neutrophil apoptosis and efferocytosis for mucosal homeostasis [111]. The probiotics have access to directly interact with the immunocompetent cells at the intestine, including within Payer’s patches, crypts and lymph nodes [112]. The probiotics at these sites are potentially capable of modulating apoptosis in immune cells. The inflamed mucosa and *Lactobacillus casei*, when cocultured, showed lowered IL-6 release, which was found to be in correlation with increased apoptotic lymphocyte proportion [113]. Similarly, *L. brevis* has been shown to induce immune cell apoptosis [114]. Sustrova et al. have demonstrated the effect of *B. bifidum, L. rhamnosus* and *E. faecium* on neutrophils to be apoptosis-inducing [115]. On the contrary, Saxami et al. (2017) showed significant inhibition of Caco-2 cell growth and proliferation upon *L. pentosus* B281 and *L. plantarum* B282 treatment; however, these strains, in a mouse model, led to rapid recruitment of immune cells, mostly comprising neutrophils among the leukocytes [116]. These results clearly indicate the species-specific activity of probiotics, strengthening the fact that each probiotic has unique functions, thereby requiring detailed studies.

### 5.3. Probiotics Influencing Neutrophil-Mediated Metastasis

Neutrophil-mediated metastasis is dependent on various tumor microenvironment factors. In this regard, with limited reports correlating the effects of probiotics on neutrophils, it is conceivable to indirectly link probiotic activity on neutrophils via these tumor microenvironment factors. Shinnoh et al. have reported induced TRAIL release from PMNs upon *Clostridium butyricum* MIYAIRI 588 (CBM588) (probiotic) administration in bladder cancer. The release mechanism of TRAIL from PMNs is not clear; however, MMP-8 is reported to be a key player in TRAIL release [95] and did not enhance TRAIL synthesis. In a previous study, *Clostridium butyricum* was reported to increase the number of CD8+ T cells and NK cells in lung cancer patients, indicating their promoted proliferation to a cytotoxic end [117]. The number of NK cells increases with age, but their functionality is lowered [118,119,120]. Intravenous injection of *L. casei* YIT9018 protected the metastatic melanoma-bearing C57BL/6 mice against pulmonary metastasis, and this was augmented with NK cell activity along with auxiliary lymph node cell cytolysis [121]. Although not studied with respect to neutrophils, the previously mentioned recent study shows the influence of active NK cells on neutrophil activity. In another study, *Lactobacillus brevis* enriched with selenium nanoparticles was found to be capable of reducing liver metastasis in a metastatic mouse (BALB/c) breast cancer model. The study is of relevance to neutrophils since the immune responses are in the form of elevated IFN-γ and IL-17 cytokine levels and enhancement of NK cell activity [122]. Similarly, *L. casei* Shirota (LcS) has shown augmentation of NK cell cytotoxicity [123].

**Figure 5:**
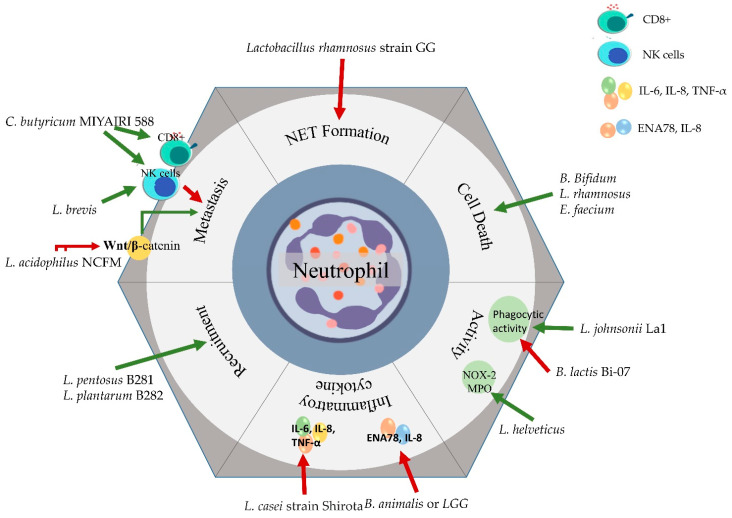
Effect of probiotics on neutrophil activity in cancer condition. Key: red arrows indicate inhibition and green arrows indicate promotion.

Through regulation of neutrophils in terms of production, function and apoptosis, the microbiome and thus the probiotics stand a chance to eventually influence the immune system [124]. Vong et al. (2014) deduced the inhibitory activity of *Lactobacillus rhamnosus* strain GG against NET formation induced by PMA and *Staphylococcus aureus*. *Lactobacillus rhamnosus* strain GG also dampened the ROS by exhibiting antioxidant activity, overall protecting against cell cytotoxicity [125]. As mentioned previously, EMT promotes migratory capacity and invasiveness in cancer cells via stromal cell-derived factor 1 (SDF-1) mediated with its receptor CXCR4 through the Wnt/β-catenin pathway. *L. acidophilus* NCFM has been reported to exert antimetastatic effects by downregulating CXCR4 expression in the spleen, colon and mesenteric lymph nodes of tumor-bearing mice [126]. As chemoprotective modulators, *L. casei* CRL431 and *L. rhamnosus* CRL150 showed the ability to increase the number of immature myeloid progenitors in the bone marrow to allow recovery of myeloid cells after cyclophosphamide treatment in mice [96]. Thereby, probiotic supplementation can influence the adaptive feature development attributed to neutrophils along with other immune cells [127]. The effect of probiotics on neutrophil activity in cancer is summarized in Figure 5.

## 6. Concluding Remarks and Future Directions

The role of neutrophils and the associated NET formation in disease conditions such as cancer is relatively emerging and assertively requires more attention. This review represents an update on the metastatic front of neutrophil activity and puts forward a comprehensive overview of probiotics as a potential therapeutic approach. Although limited, studies indicate the strain specificity of the probiotics towards neutrophil recruitment, indicating an advanced need for thorough examination of the direct and indirect roles of probiotics in cancer via neutrophils.

## Data Availability

Not applicable.
